# Serum iron levels in tuberculosis patients and household contacts and its association with natural resistance‐associated macrophage protein 1 polymorphism and expression

**DOI:** 10.1111/crj.13677

**Published:** 2023-08-22

**Authors:** Wiendra Waworuntu, Francisca Srioetami Tanoerahardjo, Anwar Mallongi, Ahyar Ahmad, Muhammad Amin, Irawaty Djaharuddin, Agussalim Bukhari, Nur Ahmad Tabri, Burhanuddin Bahar, Najdah Hidayah, Handayani Halik, Muhammad Nasrum Massi

**Affiliations:** ^1^ Pusat Kebijakan Sumber Daya dan Sistem Ketahanan Kesehatan, Badan Kebijakan Pembangunan Kesehatan Ministry of Health Republic Indonesia Jakarta Indonesia; ^2^ Postgraduate Program, Faculty of Medicine, Universitas Hasanuddin Makassar South Sulawesi Indonesia; ^3^ Department of Environmental Health, Faculty of Public Health Universitas Hasanuddin Makassar South Sulawesi Indonesia; ^4^ Department of Chemistry, Faculty of Mathematics and Natural Sciences Universitas Hasanuddin Makassar South Sulawesi Indonesia; ^5^ Department of Pulmonology and Respiratory Diseases, Faculty of Medicine Universitas Airlangga Surabaya West Java Indonesia; ^6^ Department of Pulmonology and Respiratory Diseases, Faculty of Medicine Universitas Hasanuddin Makassar South Sulawesi Indonesia; ^7^ Dr. Wahidin Sudirohusodo Hospital Makassar South Sulawesi Indonesia; ^8^ Department of Clinical Nutrition, Faculty of Medicine Universitas Hasanuddin Makassar South Sulawesi Indonesia; ^9^ Department of Nutrition Sciences, Faculty of Public Health Hasanuddin University Makassar Indonesia; ^10^ Research Center for Vaccine and Drugs National Research and Innovation Agency (BRIN) Tangerang Selatan Banten Indonesia; ^11^ Hasanuddin University Medical Research Center Laboratory, Faculty of Medicine Universitas Hasanuddin Makassar South Sulawesi Indonesia; ^12^ Department of Clinical Microbiology, Faculty of Medicine Universitas Hasanuddin Makassar South Sulawesi Indonesia

**Keywords:** gene expression, genetic polymorphism, household, iron deficiency anemia, natural resistance‐associated macrophage protein 1, tuberculosis

## Abstract

**Background:**

Iron deficiency can impair immune function, increasing tuberculosis (TB) susceptibility and severity. The research aimed to investigate iron deficiency anemia in TB patients and household contacts and its association with natural resistance‐associated macrophage protein 1 (NRAMP1) polymorphism and expression.

**Methods:**

The levels of iron, ferritin, and transferrin were measured in the serum by ELISA (Enzyme‐Linked Immunosorbent Assay). *NRAMP1* polymorphisms were determined by polymerase chain reaction (PCR) and sequencing. *NRAMP1* gene expression was measured by real‐time PCR. Interferon‐gamma release assay (IGRA) checked on household contacts to screen household contacts with positive IGRA as the control.

**Results:**

This study involved 35 TB cases and 35 TB contacts. The results showed that the serum Fe levels were found to be lower in the TB case group (median 149.6 μmol/L) than in the positive IGRA household contacts group (median 628.53 μmol/L) with a *p*‐value <0.001. Meanwhile, ferritin levels in TB cases tended to be higher, in contrast to transferrin, which was found to tend to be lower in TB cases than household contacts but did not show a significant difference. This study found no association between the polymorphism of exon 15 D543 and active TB. However, *NRAMP1* gene expression was lower in TB cases than in positive IGRA household contacts (*p* = 0.011). Besides, there was a positive correlation between *NRAMP1* gene expression and serum Fe levels (r = 0.367, *p* = 0.006). TB was associated with decreased *NRAMP1* gene expression (OR 0.086 95% CI 0.02–0.366, *p* = 0.001). Besides, TB was associated with low Fe levels (OR 0.533 95% CI 0.453–0.629, *p* < 0.001).

**Conclusion:**

Comparing the TB case to the household contacts group, decreased serum Fe levels were discovered in the TB case group. This study also shows a correlation of *NRAMP1* gene expression to Fe levels in TB patients and household contacts and describes that TB may lead to decreased Fe levels by downregulating *NRAMP1* expression.

## Graphical abstract

*M. tuberculosis* needs to obtain iron to survive and grow inside its host. *M.tuberculosis* suppresses ferroportin expression, which increases Fe levels in the phagosome. It also obtains Fe by increasing transferrin receptor (TFR) expression. These conditions cause a decrease in Fe in the blood, and thus anemia occurs. Besides, IFN‐gamma, which releases upon immune activation, downregulates the expression of TFR. The ability of NRAMP1 and FPN1 to remove iron from the phagolysosome limits the availability of intracellular iron to intracellular bacteria. Alteration in the *NRAMP1* gene may result in a nonfunctional protein or decrease the expression of *NRAMP1*, leading to more active replication of the bacteria in lung alveolar macrophage. This study demonstrated that TB was associated with decreased *NRAMP1* gene, *NRAMP1* gene expression was correlated to Fe levels, and TB was associated with low Fe levels. This finding would suggest that TB can lower Fe levels by downregulating NRAMP1 expression.

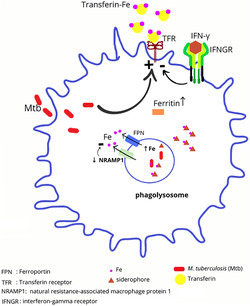

## 1 INTRODUCTION

Tuberculosis is still a global public health concern since it is the second most deadly infectious disease after COVID‐19 worldwide.^1^ *Mycobacterium tuberculosis (Mtb)* the causative agent, is estimated to infect about 1 in 25 people worldwide.^2^ Anemia is a common extra comorbidity in TB patients and negatively impacts treatment outcomes.^3^, ^4^ In Indonesia, the third largest contributor to cases, anemia accounts for 50%–70% of tuberculosis patients.^1^, ^5^, ^6^ Although the causes of anemia in tuberculosis are diverse, anemia of inflammation typically has a significant impact.^7^, ^8^ Anemia is a common complication of TB, a chronic inflammatory illness. Anemia in pulmonary TB patients may be brought on by pyridoxine deficiency, hemoptysis, blood loss, anemia of inflammation, and other factors such as side effects of anti‐tuberculous treatment.^5^

Iron deficiency may increase susceptibility to various infectious diseases since macrophages need iron to function properly.^9^ On the other hand, the innate immunological response of hypoferremia during infection is to withhold iron from pathogens through iron sequestration and reduced food absorption.^10^ Like many pathogens, *Mtb* needs to obtain iron to survive and grow inside its host.^11^ *Mtb* in cells tries to get iron in various ways. *Mtb* produces siderophores to chelate cytoplasmic iron. *Mtb* can modify host cell iron homeostasis to boost iron availability. *Mtb* suppresses ferroportin expression, which increases Fe levels in the phagosome. Some intracellular microorganisms obtain Fe by increasing transferrin receptor (TFR) expression.

Iron deficiencies and overload in tuberculosis are linked to the disease's progress and unfavorable clinical results.^3^ In TB patients, low plasma ferritin predicted an increased independent risk of treatment failure, mortality, and recurrence among HIV‐infected patients. On the other hand, high plasma ferritin was found to raise the risk of TB death.^3^ Approaches to maintaining proper iron status levels in TB patients may be beneficial in lowering TB morbidity and death. However, due to the confounding effects of inflammation on commonly used iron status biomarkers like ferritin and soluble TFR, it is challenging to discriminate between anemia of inflammation and iron‐deficiency anemia in tuberculosis patients. Besides, iron supplementation may exacerbate tuberculosis since *Mtb* requires iron for essential metabolic pathways.^12^ Thus, deciding whether (and when) to administer iron to anemic tuberculosis patients is challenging.

Genetic factors have been reported to play a role in regulating iron metabolism according to the needs of *Mtb*. *Mtb* requires iron and stores it; the role of ferritin on iron homeostasis during infection is still not well understood.^13^, ^14^, ^15^ *Natural resistance‐associated macrophage protein 1 (NRAMP1)* gene is known to encode an essential metal transporter protein that plays a role in the entry of Fe^2+^, Mn^2+^, and other metal ions, which occur in phagocytic cells.^16^, ^17^, ^18^, ^19^, ^20^ Human gene variations such as the *NRAMP1* polymorphism increase Fe levels in macrophages, increasing the risk of TB and worsening disease outcomes. The NRAMP1 protein has a dual function for carrying Fe into and out of the phagosome cell, which is affected by the pH of the cell.^21^, ^22^, ^23^ Single nucleotide polymorphism (SNP) found in four specific regions of the *NRAMP1* gene has been extensively studied and is associated with heterogeneity, gene function as the Fe transporter, and susceptibility to TB disease. *NRAMP1* polymorphism is influenced by ethnicity in susceptibility to TB disease.^17^, ^24^, ^25^, ^26^, ^27^, ^28^, ^29^

The results of the *NRAMP1* polymorphism meta‐analysis concluded that there was a correlation with susceptibility to the occurrence of pulmonary TB disease.^29^ The role of Fe in the pathogenesis of TB is prominent, especially during the initial infection and in chronic conditions. However, the analysis of *NRAMP1* polymorphisms, its expression and Fe levels in TB cases and their household contacts is still lacking. It is expected that the assessment of variations in genetic factors that influence the occurrence of diseases associated with the role of genes in specific research designs in airborne disease transmission will be useful for policymakers.

We studied iron deficiency anemia (IDA) in TB patients in the high‐incidence setting of TB in Indonesia. We investigated other parameters related to iron homeostasis, that is, ferritin and transferrin levels, and compared these parameters with people in contact households who were latently infected. We also investigated the association between TB, *NRAMP1* gene expression and Fe levels.

## 2 MATERIAL AND METHODS

### 2.1 Participants selection and sample collection

This research is a case–control study. TB case samples were patients who were registered at the Makassar Community Lung Health Center (BBKPM) during the study period. Then, the patient and their household contacts were asked about his willingness to participate in this study. Active TB patients recruited were new TB case patients with no history of previous TB drugs, with active TB symptoms, positive smear examination, and HIV‐negative results, and were willing to be contacted further to trace contacts at home. Meanwhile, the household contacts included were contacts who lived at the same house with active TB patients for at least 2 weeks, did not show any active TB symptoms, had no history of previous TB therapy, and had positive IGRA results. TB patients and household contacts were interviewed using a questionnaire. Venous blood samples were obtained for all participants, and sputum samples were specifically for TB patients. This research was conducted at the HUM‐RC Laboratory (Hasanuddin University Medical—Research Center) Faculty of Medicine, Hasanuddin University, Makassar.

This study complies with the Declaration of Helsinki and has been approved by the Health Research Ethics Committee of the Faculty of Medicine, Universitas Hasanuddin (115/UN4.6.4.5.31/PP36/2023). Each subject gave written informed consent and agreed to participate in the study.

### 2.2 Interferon‐gamma release assay

Interferon‐gamma (IFN‐γ) release assay (IGRA) was performed in household contacts by using QuantiFERON Gold Plus TB Test kit (QFT‐Plus, Qiagen, Germany). Six mL of venous blood was obtained from participants and transferred 0.8–1.2 mL to 4 QFT‐Plus tubes (Nil, TB1, TB2, Mitogen). Four tubes were simultaneously shaken until covered in blood. These four tubes were incubated at 37°C for 16–24 h, followed by a 15‐min centrifugation to separate the plasma. Plasma samples were stored at −20 C until employed in the IFN‐γ ELISA (enzyme‐linked immunosorbent assay) procedure, according to manufacture protocols. The Optical Density (OD) was measured using an ELISA reader with a 450 nm filter and 620 to 650 nm reference filter. QuantiFERON‐TB Gold Plus Analysis Software ver. 2.71 (Qiagen) was used to calculate IFN‐γ from OD readings. A Nil value ≤8 IU/mL and a Mitogen‐Nil difference ≥0.5 IU/mL indicate that all samples were valid. The difference value of Tb1‐Nil or Tb2‐Nil ≥0.35 IU/mL was considered positive.^30^

### 2.3 Iron, ferritin, and transferrin levels measurement

The levels of iron, ferritin, and transferrin were measured in the serum by ELISA (Enzyme‐Linked Immunosorbent Assay) using Iron Microplate Assay Kit, Human Ferritin ELISA Kit, and Human Transferrin ELISA (MyBioSource, Southern California, San Diego, USA), respectively, following the manufacturer's specification. Absorbance was measured at 450 nm, with the correction wavelength set at 540 nm using a microplate reader. The concentration of iron, ferritin, and transferrin was calculated using linear regression analysis of absorbance values obtained for each measurement.

### 2.4 *NRAMP1* polymorphism

#### 2.4.1 DNA extraction

A total of 150 μL of blood sample (whole blood) was homogenized with 20 μL of Proteinase K and then incubated at 60°C for 5 min. DNA was extracted from the sample using the gSYNC™ DNA Extraction Kit (Geneaid, Taiwan). Prior to using it in PCR, the DNA extract was kept at −80°C.

#### 2.4.2 NRAMP1 gene amplification and sequencing

Analysis of the *NRAMP1* gene polymorphism was carried out by PCR, followed by the sequencing process. PCR amplification was performed using a Thermal cycler device (Bio‐Rad, USA). PCR amplification was performed using a Thermal cycler device (Bio‐Rad, USA). A total of 200 ng of template DNA was amplified with 25 ul of Kapa Biosystem Taq Polymerase Enzyme (Roche, USA), Forward and Reverse primers (listed in Table S1), and nuclease‐free water to a total reaction volume of 50 μl. After being electrophoresed on a 2% agarose gel, the PCR result was then observed under a UV light using a Gel Doc™ XR device (Bio‐Rad, USA). PCR products that have been confirmed by electrophoresis are then sent for sequencing in Malaysia's 1st Base Laboratory for direct sequencing (Sanger sequencing). The sequencing results were then aligned to the reference gene in the NCBI database using Unipro UGENE v. 44.0 (Unipro, Novosibirsk, Russia).

### 2.5 *NRAMP1* expression

The *NRAMP1* gene expression was determined by the real‐time quantitative PCR (qPCR) method using SsoFast™ EvaGreen® Supermix (Bio‐Rad, California, USA). Before amplification, the reaction mixture was prepared by adding 10 μL Eva green mix, 0.5 μL cDNA (150 ng/μL), 0.5 μL forward primer, 0.5 μL reverse primer (10 pmol each primer), and nuclease‐free water up to 20 μL. The same procedure was carried out for the GAPDH gene as a reference gene. The primers used have been described in Supporting Information, Table S2. qPCR was performed in the following conditions: 50°C for 2 min, 95°C for 1 min, 40 cycles of denaturation at 95°C for 15 s then followed by annealing 60°C for 30 s and 72°C extension for 30 s. The last cycle was the final extension at 72°C for 10 min.

### 2.6 Statistical analysis

Categorical data were compared using the chi‐square test or Fischer‐exact test. The *t*‐test or Mann–Whitney *U*‐test and One‐way ANOVA or Kruskall‐Wallis were used to compare the value difference between two and three groups, respectively. Spearman's rank correlation test was used to determine the correlation between serum iron levels and *NRAMP1* expression. SNPStats online software (https://www.snpstats.net) was used to determine the deviation of *NRAMP1* polymorphisms from the Hardy–Weinberg equilibrium, measure the genotype frequencies, and assess the association of *NRAMP1* genotype with TB cases using household contacts as the control group. *NRAMP1* relative gene expression was obtained using the software Bio‐Rad CFX™ Manager (v.3.1, Bio‐Rad Lab. Inc., Hercules, CA, USA). A generalized linear model was performed to investigate the relationship between the independent and dependent variables. A *p*‐value less than 0.05 was considered significant.

## 3 RESULTS

### 3.1 Participant characteristics

**FIGURE 1:**
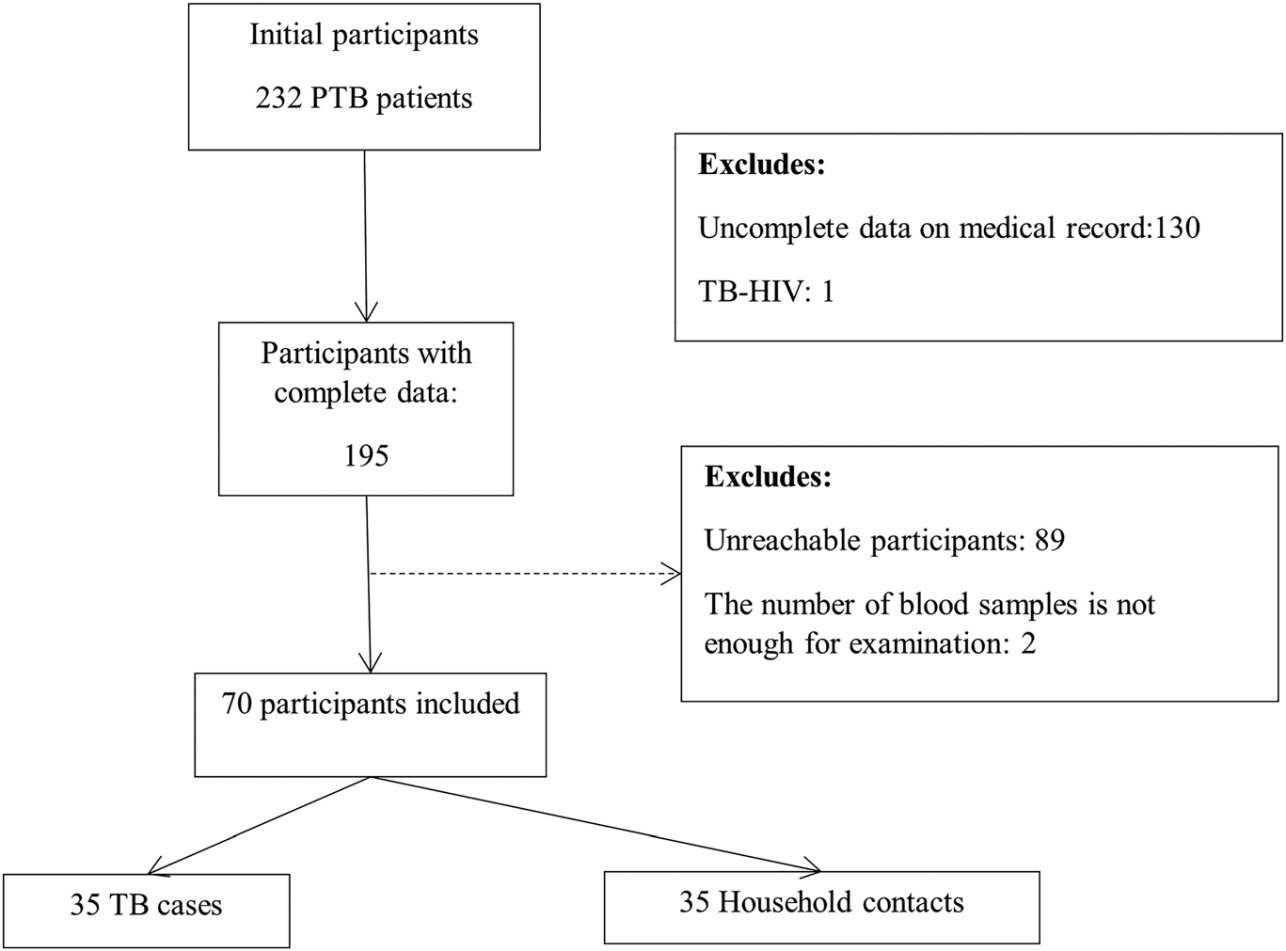
Participants diagram.

The research participants collected during the study period were 35 TfB cases and 35 TB positive IGRA contact cases at home. The data of the research subjects (overall) of 328 respondents were made in a diagram in stages according to the examinations carried out so that 70 cases would be obtained where the biomarker data of iron metabolism were all complete (Figure 1).

The results of the analysis of the characteristics of the respondents are shown in Table 1.

**TABLE 1:** Characteristics of participants.

Characteristics	Active TB (*N* = 35)	Latent TB (*N* = 35)	*p*‐Value
Age, no. (%)			0.629
≤39 years old	14 (40%)	16 (45.7%)	
>39 years old	21 (60%)	19 (54.3%)	
Sex, no. (%)			0.055
Female	15 (42.9%)	23 (65.7%)	
Male	20 (57.1%)	12 (34.3%)	
BMI, no. (%)			0.003*
<18.5 kg/m^2^	18 (54.1%)	6 (17.1%)	
≥18.5 kg/m^2^	17 (48.6%)	29 (82.9%)	
Smoker, no. (%)			0.007*
Yes	19 (54.3%)	8 (22.9%)	
No	16 (46.7%)	27 (77.1%)	
Diabetes Mellitus, no. (%)			
Yes	4 (11%)	0 (0%)	
No	31 (89%)	35 (100%)	
Relation to index cases, no. (%)			
Spouse		24 (62%)	
Parent/son/daughter		3 (8%)	
Siblings			
Others		2 (5%)	

* Significant *p*‐value: *p* < 0.05.

### 3.2 Hemoglobin (Hb) measurement

In this study, Hb levels in the TB case group were found to be lower (median 11.9 g/dL) than the household contact group (median 13 g/dL) with a *p* = 0.013, as shown in Table 2). Because there were differences in determining anemia status in men and women, the analysis was continued by sex grouping. Similar results were shown for both male and female respondents, where Hb levels were significantly lower in the TB case group (*p* = 0.017 and *p* = 0.033).

**TABLE 2:** Hemoglobin (Hb) levels in the TB case group and household contacts.

	Sex	TB cases	Household contacts	*p*‐Value
Hb (gr/dL), median (minimum–maximum)	All sex	11.9 (1.9–18)	13 (9.3–16.8)	0.013
	Male	12.9 (9.2–18)	13.95 (12.5–16.8)	0.017
	Female	11.7 (1.9–14)	12.4 (9.3–14.1)	0.033

Based on WHO criteria regarding anemia as stated in this study's operational definition and objective criteria, we analyzed the frequency of respondents experiencing anemia in both the TB group and household contacts. The Chi‐square test results showed that the proportion of anemia respondents was higher in the TB case group (62.1%) compared to the household contact group (30.3%) with a *p* = 0.012.

### 3.3 Iron, ferritin, and transferrin levels measurement

In this study, serum Fe levels were found to be significantly lower in the TB case group (median 149.6 μmol/L) than in the household contacts group (median 628.53 μmol/L) with a *p*‐value <0.001. Meanwhile, ferritin and transferrin levels did not differ significantly between the TB case groups and household contacts, with *p*‐values of 0.184 and 0.307, respectively (Table 3).

**TABLE 3:** Serum levels of Fe, ferritin, and transferrin in the TB case group and household contacts.

Measurements	TB cases	Household contacts	*p*‐Value
Serum Fe (μmol/L), median (minimum–maximum)	149.6 (4.43–717.51)	628.53 (268.3–1947.8)	<0.001*
Ferritin (ng/mL), median (minimum–maximum)	111.8 (99.9–131.73)	115.1 (97.1–131.73)	0.184
Transferrin (ng/mL), mean ± SD	60.96 ± 25.2	61.59 ± 30.6	0.307

* Significant *p*‐value: *p* < 0.05.

### 3.4 *NRAMP1* polymorphism

The genotype frequencies of the *NRAMP1* polymorphism exon 3‐274 C/T (rs2276631), intron 4469 + 14 G/C (rs3731865), and exon 15 D543N (rs17235409) in the contact group (as a control) are consistent with Hardy–Weinberg equilibrium (*p* > 0.05). In comparison, the Hardy Weinberg equilibrium for exon 4 C125R (rs748447891) and 3' UTR delTGTG (rs17235416) could not be determined because no mutation on exon 4 C125R and TGTG deletions were found in the household contact samples in this study.

The genotype frequencies of the polymorphisms examined in this study are shown in Table 4. The dominant homozygotes dominated each polymorphism examined. Meanwhile, none of the samples were found to be homozygous recessive. Polymorphism exon 15 D543N (rs17235409) heterozygous (G/A) was found in 20.6% of TB cases and 22.9% of household contacts. Meanwhile, 3' UTR delTGTG (rs17235416) analysis revealed that all samples (100%) had the TGTG/TGTG genotype. Based on the sequencing results, none of the samples detected TGTG deletion, either in the TB case group or the household contact group.

**TABLE 4:** Association of NRAMP1 polymorphisms with TB cases.

SNP	Genotype	TB cases	Household contacts (control)	OR (95% CI)	*p*‐Value
Exon 3–274 C/T (rs2276631), no. (%)^†^	C/C	31 (96.9%)	33 (97.1%)	NA	
	C/T	1 (3.1%)	1 (2.9%)		
	T/T	0 (0%)	0 (0%)		
Exon 4 C125R (rs748447891), no. (%)^‡^	T/T	31 (96.9%)	33 (100%)	NA	
	T/G	1 (3.1%)	0 (0%)		
	G/G	0 (0%)	0 (0%)		
Intron 4469 + 14 G/C (rs3731865), no.(%)^‡^	G/G	31 (96.9%)	30 (90.9%)	NA	
	G/C	1 (1%)	3 (9.1%)		
	C/C	0 (0%)	0 (0%)		
Exon 15 D543N (rs17235409), no.(%)^§^	G/G	27 (79.4%)	27 (77.1%)	1	0.82
	G/A	7 (20.6%)	8 (22.9%)	0.88 (0.28–2.75)	
	A/A	0 (0%)	0 (0%)		
3'UTR delTGTG (rs17235416), no.(%)^§^	+/+	34 (100%)	35 (100%)	NA	
	+/del	0 (0%)	0 (0%)		
	del/del	0 (0%)	0 (0%)		

Abbreviation: NA, not available.

^†^ Data available on 66 samples.

^‡^ Data available on 65 samples.

^§^ Data available on 69 samples.

This study found no association between the polymorphism of exon 15 D543 and active tuberculosis. Besides, the relationship between the *NRAMP1* polymorphism exon 3–274 C/T, intron 4 G/C, exon 4 C125R, and 3' UTR delTGTG with TB cases could not be determined in this study due to the minimal genotype variation of exon 3–274 C/T, intron 4 G/C, and exon 4 C125R, and no 3' UTR mutations were found in the samples of this study.

Analysis of the association of *NRAMP1* haplotypes with TB cases did not show any significant association between certain *NRAMP1* haplotypes and the incidence of active pulmonary TB (*p* > 0.05), as described in Table 5.

**TABLE 5:** Association analysis of NRAMP1 haplotypes with TB cases.

Exon 3−274 C/T	Exon 4 C125R	Intron 4 G/C	Exon 15 D543	Frequency	OR (95% CI)	*p*‐Value
C	T	G	G	0.846	1	Ref
C	T	G	A	0.109	0.81 (0.25–2.61)	0.73
C	T	C	G	0.022	0	1
T	T	C	G	0.015	1.03 (0.06–18.91)	0.98

*Note*: The results of the analysis are shown: combinations with a frequency >0.01.

Abbreviation: Ref, reference.

### 3.5 *NRAMP1* expression

**FIGURE 2:**
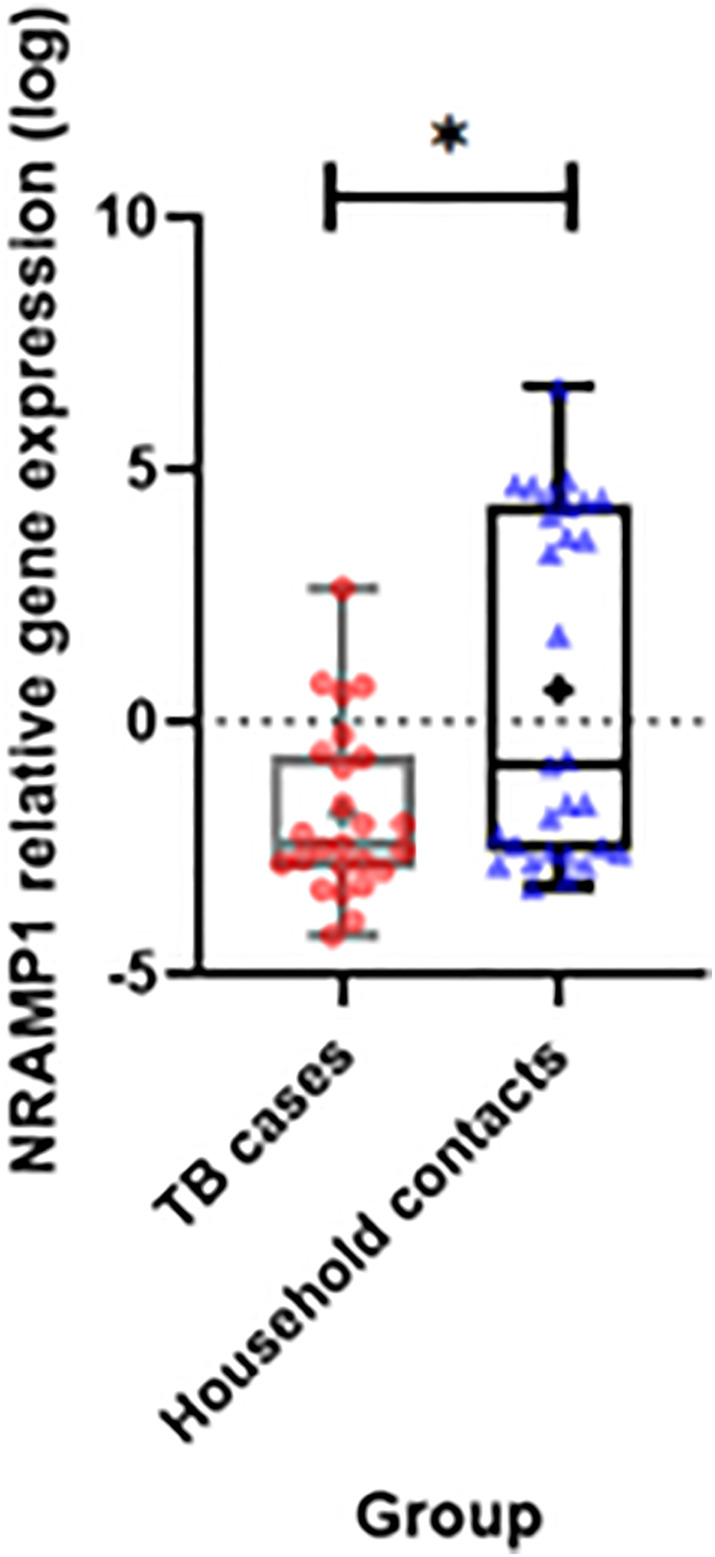
The comparison of relative expression of the NRAMP1 gene in TB case groups and household contacts. The relative expression of the NRAMP1 gene was significantly higher in the household contact group than the TB case group (*p* = 0.011). The sign (*) indicates the value of *p* < 0.05.

The results of examining the relative expression of the *NRAMP1* gene using the qPCR method showed that the relative expression of the *NRAMP1* gene was significantly higher in the contact household group than in the TB case group (*p* = 0.011) (Figure 2). The relative expression value of the *NRAMP1* gene from qPCR examination using the *GAPDH* gene as a reference gene. The relative expression ratio of the *NRAMP1* gene in TB cases compared to household contacts was 0.005: 17.317 or 1: 3463.4. This shows that the relative expression of the NRAMP1 gene was 3.463 times higher in the TB case group than in household contacts.

### 3.6 Correlation of serum Fe levels with *NRAMP1* gene expression

Using the Spearman test, Fe levels correlate positively with *NRAMP1* gene expression (r = 0.367, *p* = 0.006). This analysis showed that the higher the *NRAMP1* gene expression, the higher the serum Fe level.

### 3.7 Association between TB, *NRAMP1* gene expression, and Fe levels

Based on the generalized linear models, TB was associated with decreased *NRAMP1* gene expression (OR 0.086 95% CI 0.02–0.366, *p* = 0.001). Besides, TB was associated with low Fe levels (OR 0.533 95% CI 0.453–0.629, *p* < 0.001).

## 4 DISCUSSION

The management of iron availability is crucial under homeostasis; it becomes considerably more crucial during an infection. There are different ways to limit iron for invasive pathogens as a result of iron's essential role in the host and microbe interaction and the subsequent impact on the progression of infections. In this study, serum Fe levels were significantly lower in the TB case group, with a median of 149.6 μmol/L compared to the household contacts group, with a median of 628.53 μmol/L (*p* < 0.001). Research by Hella et al. also showed significantly lower Fe levels in TB cases than in controls.^31^ Following immune activation, diverse interactions between immune cells result in the release of a range of inflammatory mediators, including IL‐6, which inhibits transferrin production and stimulates hepcidin antimicrobial peptide (HAMP) transcription in hepatocytes. HAMP, the FPN1 ligand, binds to the gatekeeping iron exporter ferroportin‐1 (FPN1), thus reducing iron transport to the plasma and reducing the level of transferrin‐bound iron. Besides, IFN‐γ inhibits the proliferation of erythroblast, while reactive oxygen species (ROS) and immunoglobulins (IG) expand the number of damaged RBCs (dRBCs). These conditions cause a decrease in Fe in the blood, and thus anemia occurs.^32^

In this study, the median Ferritin level in TB cases was 111.8 ng/mL, while in contact cases, it was 115.1 ng/mL. WHO defines low serum ferritin in adults as 15 g/L (15 ng/mL) in the general population.^33^ The 30 μg/L limit has been reported as the highest sensitive (92%) and specific (98%) threshold for identifying iron deficiency, coinciding with the lack of iron reserves in bone marrow (despite whether or not anemia exists).^34^ In inflammatory conditions, expert recommendations often define the threshold of serum ferritin and TSAT levels for iron deficiency diagnosis,^35^, ^36^, ^37^ but cut‐off values are inconsistent.^35^ Serum ferritin is a blood protein, an acute phase reactant, and an inflammatory marker in various inflammatory diseases. Ferritin values in cases of inflammation range between 30 and 300 ng/mL in men and between 10 and 200 ng/mL in women. Increased ferritin levels in pulmonary TB patients ranged between 500 and 800 ng/dL.^38^ Higher ferritin amounts have been recorded in patients with pulmonary tuberculosis.^39^, ^40^ It has been suggested that the raised ferritin levels in TB are due to the increased production of monocytes and macrophages, which is elevated during TB infection.^41^ Ferritin levels decrease along with anti‐tuberculosis treatment, especially after 2 months of treatment.^42^ Although past studies have shown the possible utility of serum ferritin levels for TB diagnostic purposes,^43^ additional indicators of iron homeostasis, either separately or together, have not been thoroughly explored for diagnosing TB according to the disease phase.

The transferrin levels in TB cases and household contacts in this study were found to be: 60.96 and 61.59 ng/mL, but statistically, the difference in ferritin levels between groups was not significant. In the case of TB, transferrin can decrease due to suppression of synthesis caused by pro‐inflammatory cytokines such as IL‐6. During infection, the content of iron storage proteins in the human host increases, and free iron ions bind to iron‐transporters (e.g., transferrin) that proactively maintain a lower serum iron level to limit iron uptake by pathogens. The human body also decreases the absorption of iron from the intestine. Together, these mechanisms limit the iron available to pathogens and hence inhibit bacterial reproduction in the body.^44^

In this study, no polymorphism of exon 15 D543 was found. These results are in line with research in Surabaya, Indonesia.^45^ While the relationship between the *NRAMP1* polymorphism exon 3–274 C/T, intron 4 G/C, exon 4 C125R, and 3' UTR delTGTG with TB cases could not be determined in this study due to the minimal genotype variation of exon 3–274 C/T, intron 4 G/C, and exon 4 C125R, and no 3' UTR mutations were found in the samples of this study. The link between *NRAMP1* variation and tuberculosis is not general since numerous other research have shown no association between *NRAMP1* genetic variants and TB risk in other races. The different results might be attributed to variations in the research population, such as racial diversity. A meta‐analysis study attempted to investigate subgroups and found D543N G/A variant was related to a greater risk of active TB in Americans, while the intron 4 G/C variant in Africans.^29^ Moreover, these inconsistent findings may be due to the study's small number of participants and greater heterogeneity.

*NRAMP1* gene expression was decreased in TB compared to household contacts in this study. Decreased NRAMP1 may contribute to increased iron availability. NRAMP1 transports iron from macrophages to intracellular bacteria such as *Mtb*. Because iron is also necessary for cells to create reactive oxygen and intermediate nitrogen, reducing intramacrophage iron availability to bacteria while concurrently negatively impacting the antimicrobial properties of the NRAMP1 protein may assist *Mtb* infection and growth.^46^ The study of Anggraini et al. showed similar results, where *NRAMP1* expression was found to be low in TB patients compared to healthy controls (nurses as controls), and this was associated with the *NRAMP1* D543N polymorphism.^45^ Low *NRAMP1* expression may predispose individuals to develop an active TB, combined with host and other environmental variables.^47^ Additionally, *NRAMP1* influences cytokine synthesis in a variety of ways, MHC class II molecule expression, and T cell protein antigen processing.^48^ Disruption of *NRAMP1* expression may limit the pro‐inflammatory capacity of macrophages, causing the decreased formation of nitric oxide (NO), TNF‐α, and interleukin‐6, and increased synthesis of interleukin‐10.^19^

Fe levels correlate positively with *NRAMP1* gene expression (r = 0.367, *p* = 0.006). The results of this analysis indicate that the higher the expression of the *NRAMP1* gene, the serum Fe level will also increase. Fe metabolism and its transport to tissues can be impacted by low *NRAMP1* expression. Because Fe metabolism is required for macrophage function, cell‐autonomous phagosomal membrane activity combined with the impacts of ROS‐based signaling of cytoplasmic Fe may influence their inflammatory potential.^19^

This research showed that TB was associated with decreased *NRAMP1* gene, *NRAMP1* gene expression was correlated to Fe levels, and TB was associated with low Fe levels. This finding would suggest that TB can lower Fe levels by downregulating *NRAMP1* expression. Overall, this research and earlier studies' findings suggest that impeded and poor *NRAMP1* expression may impact a person's ability to successfully block the replication of *Mtb* due to decreased *NRAMP1* and, ultimately, the manifestations of active TB. The expression of specific microbial pathogenicity determinants necessary for intracellular survival may be affected by NRAMP1‐mediated reduction of divalent ion concentrations. Alternatively, the maturation process of the phagosome from the greatly bactericidal and bacteriostatic fused phagolysosome may also be affected.^49^ The ability of NRAMP1 and FPN1 to remove iron from the phagolysosome limits the availability of intracellular iron to intracellular bacteria. In addition to directly attacking intramacrophage bacteria, the labile radical NO also increases FPN1 expression, which lowers intracellular iron.^50^ Alteration in the *NRAMP1* gene may result in a non‐functional protein or decrease the expression of *NRAMP1*, leading to more active replication of the bacteria in lung alveolar macrophage.^45^, ^47^, ^51^, ^52^

There are some limitations in this research. The other possible causes of low iron levels in the participants were not investigated further in this study. Iron deficiency can be due to several factors, both absolute, such as inadequate iron intake or blood loss, or functional, such as infection or inflammatory conditions.^53^ This is one of the limitations of this study because we did not obtain information such as iron intake or whether there was a history of bleeding because we focused on measuring laboratory parameters (Fe, ferritin, and transferrin) and *NRAMP1*. Besides, this study was a case–control study in which the parameters examined, *NRAMP1* expression and serum levels of Fe, ferritin, and transferrin, were carried out once. The cohort study will provide sufficient data to follow the course of TB disease related to Fe homeostasis in TB cases and contacts (TB infection). However, the results of this case–control study can describe differences in Fe levels and lower *NRAMP1* expression in the TB group (before treatment) compared to household contacts. As far as we know, this is the first study to investigate the polymorphism and gene expression of *NRAMP1* together with Fe, ferritin, and transferrin levels in TB cases compared to household contacts with latent TB. In the future, it is necessary to conduct further research by looking at other biomarkers related to anemia and individual susceptibility to tuberculosis infection. In addition, cohort analysis is needed in TB patients to see the effect of *NRAMP1* expression and Fe, ferritin, and transferrin levels on disease outcomes and treatment, as well as in household contacts to see whether there is an effect of *NRAMP1* expression as well as Fe, ferritin, and transferrin levels on the incidence of TB reactivation.

## 5 CONCLUSION

Serum Fe levels in the TB case group were found to be lower when compared to the household contacts. This study also demonstrates a relationship between the expression of the *NRAMP1* gene and Fe levels in TB patients and household contacts, and suggests that TB may cause Fe levels to drop by downregulating the expression of *NRAMP1*.

## AUTHOR CONTRIBUTIONS

**Wiendra Waworuntu**: Conceptualization; investigation; formal analysis; writing original draft. **Francisca Srioetami Tanoerahardjo**: Conceptualization; supervision; methodology; validation; writing—review and editing. **Anwar Mallongi**: Conceptualization; supervision; methodology; validation; writing—review and editing. **Ahyar Ahmad**: Methodology; validation; writing—review and editing. **Muhammad Amin**: Validation; writing—review and editing. **Irawaty Djaharuddin**: Methodology; validation; writing—review and editing. **Agussalim Bukhari**: Methodology; validation; writing—review and editing. **Nur Ahmad Tabri**: Methodology; validation; writing—review and editing. **Burhanuddin Bahar**: Methodology; validation; writing—review and editing. **Najdah Hidayah**: Investigation; formal analysis; writing original draft. **Handayani Halik**: Investigation; formal analysis; writing original draft. **Muhammad Nasrum Massi**: Conceptualization; supervision; methodology; validation; writing—review and editing. All authors discussed the results and contributed to the final manuscript.

## CONFLICT OF INTEREST STATEMENT

There are no disclosed conflicts of interest for the authors. The manuscript's contents have been reviewed and approved by each co‐author, and there are no competing financial interests to state. We confirm that the submission is unique and is not already being considered by another publisher.

## ETHICS APPROVAL STATEMENT

This study has been approved by Health Research Ethics Committee of Faculty of Medicine, Universitas Hasanuddin (115/UN4.6.4.5.31/PP36/2023). Every participant in the study provided signed, fully informed consent.

## Supporting information


**Data S1** Supporting Information

## Data Availability

The study's supporting data are accessible upon request from the corresponding author.
