# Supplementary material for: Serum iron levels in tuberculosis patients and household contacts and its association with natural resistance‐associated macrophage protein 1 polymorphism and expression
Source: Clin Respir J. 2023 Aug 22;17(9):893–904. doi: 10.1111/crj.13677 (PMC10500328; doi:10.1111/crj.13677)
Supplement: Supplementary file 1 — Data S1 Supporting Information [file CRJ-17-893-s001.docx]

SUPPLEMENTARY MATERIALS

**Table 1. Primers used in NRAMP1 polymorphisms**

| ***NRAMP1*** | **Primer** |
| --- | --- |
| **Exon 3**  **-274 C/T**  (rs2276631) | F:  5’-TGC CAC CAT CCC TAT ACC CAG-3’  R:  5’-TCT CGA AAG TGT CCC ACT CAG-3’ |
| **Intron 4**  **469+14 G/C**  (rs3731865) and  **Ekson 4 C125R**  (rs748447891) ** | F:  5′-CTCCCTGCCTCCTCACAGCTTCT-3′,  R:  5′-CTTGGGATGCCCCATGTGAGA-3′ |
| **Exon 15** (rs17235409) D543N G>A 17981 and  **3’ UTR**  **TGTG +/del)** (rs17235416) | F:  5′-AACTGTCCCACTCTATCCTG-3′  R:  5′-GGCATCTCCCCAATTCATGGTTG-3′, |

**Table 2. Primers used in real-time PCR**

| **Target** | **Primer** |
| --- | --- |
| ***NRAMP1*** | F:  5’-GCATCTCCCCAATTCATGGT-3’  R:  5’- CAGGATAGAGTGGGACAGTT-3’ |
| **GAPDH (reference gene)** | F:  5’-CCTGCACCACCAACTGCCTTA-3’,  R:  5′- GGCCATCCACAGTCTTCTGAG-3′ |
